# Social isolation in the oldest-old: determinants and the differential role of family and friends

**DOI:** 10.1007/s00127-023-02524-x

**Published:** 2023-07-05

**Authors:** Katharina Isabelle Moormann, Alexander Pabst, Franziska Bleck, Margrit Löbner, Hanna Kaduszkiewicz, Carolin van der Leeden, André Hajek, Christian Brettschneider, Kathrin Heser, Luca Kleineidam, Jochen Werle, Angela Fuchs, Dagmar Weeg, Horst Bickel, Michael Pentzek, Siegfried Weyerer, Birgitt Wiese, Michael Wagner, Wolfgang Maier, Martin Scherer, Hans-Helmut König, Steffi G. Riedel-Heller

**Affiliations:** 1 Institute of Social Medicine, Occupational Health and Public Health (ISAP), Faculty of Medicine, University of Leipzig, Philipp-Rosenthal-Straße 55, 04103 Leipzig, Germany; 2 Department of Primary Medical Care, Center for Psychosocial Medicine, University Medical Center Hamburg-Eppendorf, Hamburg, Germany; 3 Department of Health Economics and Health Services Research, Hamburg Center for Health Economics, University Medical Center Hamburg-Eppendorf, Hamburg, Germany; 4 Department of Neurodegenerative Diseases and Geriatric Psychiatry, University Hospital Bonn, Bonn, Germany; 5 Central Institute of Mental Health, Medical Faculty Mannheim, Heidelberg University, Heidelberg, Germany; 6 Institute of General Practice, Medical Faculty, Heinrich Heine University Düsseldorf, Düsseldorf, Germany; 7 Department of Psychiatry, Technical University of Munich, Munich, Germany; 8 Institute of General Practice and Primary Care, Chair of General Practice II and Patient Centredness in Primary Care, Faculty of Health/School of Medicine, Witten/Herdecke University, Witten, Germany; 9 Institute of General Practice, Hannover Medical School, Hannover, Germany

**Keywords:** Social isolation, Social network, Family, Friends, Cohort study, Oldest-old, Risk factor, Prevalence

## Abstract

**Purpose:**

To examine the association of sociodemographic and health-related determinants with social isolation in relation to family and friends in the oldest-old.

**Methods:**

Database was the multi-center prospective AgeCoDe/AgeQualiDe cohort study assessed at follow-up wave 5 (*N* = 1148; mean age 86.6 years (SD 3.0); 67% female). Social isolation was assessed using the short form of the Lubben Social Network Scale (LSNS-6). The LSNS-6 contains two sets of items establishing psychometrically separable subscales for isolation from family and friends (ranges 0–15 points), with lower scores indicating higher isolation. Cross-sectional linear (OLS) regression analyses were used to examine multivariate associations of sociodemographic and health-related determinants with social isolation from family and friends.

**Results:**

Overall, *n* = 395 participants (34.6%) were considered socially isolated. On average, isolation was higher from friends (mean 6.0, SD 3.8) than from family (mean 8.0, SD 3.5). Regression results revealed that in relation to family, males were more socially isolated than females (β = − 0.68, 95% CI − 1.08, − 0.28). Concerning friends, increased age led to more isolation (β = − 0.12, 95% CI − 0.19, − 0.05) and functional activities of daily living to less isolation (β = 0.36, 95% CI 0.09, 0.64). Independent of the social context, depression severity was associated with more social isolation, whereas cognitive functioning was associated with less social isolation.

**Conclusions:**

Different determinants unequally affect social isolation in relation to family and friends. The context of the social network should be incorporated more strongly regarding the detection and prevention of social isolation to sustain mental and physical health.

## Introduction

Increasing life expectancy worldwide accounts for a steadily increasing proportion of older people in the demographic composition of Western societies. In Germany, the percentage of people over 80 years of age is estimated to triple between 2040 and 2060, as compared to 2008 [1]. Therefore, it is of crucial importance to pay attention especially to the concerns, challenges and needs of the oldest-old.

Social isolation is one of the major challenges for older adults due to decreasing economic and social resources, impaired mobility and the death of spouses and friends, all leading to a reduction in older people’s network size [2]. Socially isolated older individuals in particular face a variety of challenges that can adversely affect their mental and physical health [3]. A wide range of concomitant diseases are associated with social isolation, including depression [4], cardiovascular diseases [5], and cognitive decline [6], as well as a higher risk of chronic illnesses [7], suicidal behaviour [8] and premature mortality [9]. It has been argued that social isolation as a risk factor for mortality is comparable to the deleterious effects of smoking and greater than the risk of obesity [9].

Research on social integration as the opposite of social isolation corroborates these findings, indicating that positive social ties and support have beneficial influences on mental and physical health among older adults. For example, social relationships and support are associated with greater life satisfaction and self-esteem [10] and prevent depression after a loss experience and bereavement [11]. The significance of social integration has become even more important as a result of the COVID-19 pandemic [12].

Two main social network types and sources of integration among older adults are family and friends [13]. The social networks of family and friends vary in their structural and functional characteristics, quality and quantity [14]. The quantity of a social network displays aspects such as the size of social network and the frequency of contacts, the quality includes closeness, satisfaction and expectations of the relationship [15]. Friendships are made voluntarily, usually based on common interests and life stages, and they are likely to include people of the same age and provide companionship. In contrast, family relationships are obligatory, based on family history, and might be maintained partly because of cultural norms and formal obligations [16].

Multiple studies state that the quality of a social network is more strongly correlated with social isolation than the quantity [10, 14, 17]. A good quality of relationships was associated with well-being and physical health, whereas the quantity of relationships had little influence on physical and psychological health [14].

Nevertheless, the quantity of social contact with friends was more closely related to well-being than the quantity of contact with adult children [10]. This is consistent with evidence suggesting that friendships may contribute more to well-being than family relationships [14]. The absence of family in the context of friends was less detrimental than the absence of friends in the context of family. For instance, the friend’s network seems to be more beneficial than the family network in terms of physical but not mental health [18].

In sum, there is evidence that the social network of friends and family may vary among older adults and that social isolation resulting from any of these sources may have different effects on physical and mental health. At the same time, prevalence figures on social isolation in the particularly vulnerable group of oldest-olds (80+ years) are lacking [19], specifically with regard to the differentiation of isolation from family and friends. In addition, there is a lack of knowledge about context-specific determinants of social isolation, which are fundamental to prevention efforts to maintain physical and mental health among older adults. In this study, we attempt to address these gaps by (1) estimating the prevalence of social isolation from family and friends in a group of older adults, and (2) assessing the impact of sociodemographic and health-related determinants on social isolation from family and friends in this subgroup.

## Methods

### Study design and sample

Data analysis is based on general practitioner (GP) patients who participated in the longitudinal, prospective studies *German study on ageing, cognition and dementia in primary care patients * (AgeCoDe) and *Needs, health service use, costs and health-related quality of life in a large sample of oldest-old primary care patients (85+) * (AgeQualiDe). The AgeQualiDe study is a continuation (follow-ups 7–9) and extension of the AgeCoDe study.

Patients were recruited in six cities throughout Germany including Hamburg, Bonn, Düsseldorf, Leipzig, Mannheim and Munich via general practitioners. Selection requirements for participation in the baseline study were 75+ years of age, no dementia according to the GP’s diagnosis and at least one contact with the GP during the last 12 months. In Germany, approximately 94% of people above 65 years regularly seek medical advice from their GP [20]. Therefore, participants can be considered representative of the majority of community-dwelling older individuals. Following the recruitment, participants were contacted by the study centers and asked to complete the baseline assessment.

Data collection of the AgeCoDe/AgeQualiDe study took place between 2003 and 2017 including nine follow-up assessments that were scheduled approximately every 18 months. This study includes data from follow-up wave 5 only, conducted in 2011, since data on social isolation was not assessed in earlier waves. Data collection took place in the participants’ homes by trained research assistants.

The AgeCoDe/AgeQualiDe Study was conducted in accordance with the ethical standards embodied in the Declaration of Helsinki of 1975 and was approved by the local ethics committees of the participating study sites in Germany.

**Fig. 1:**
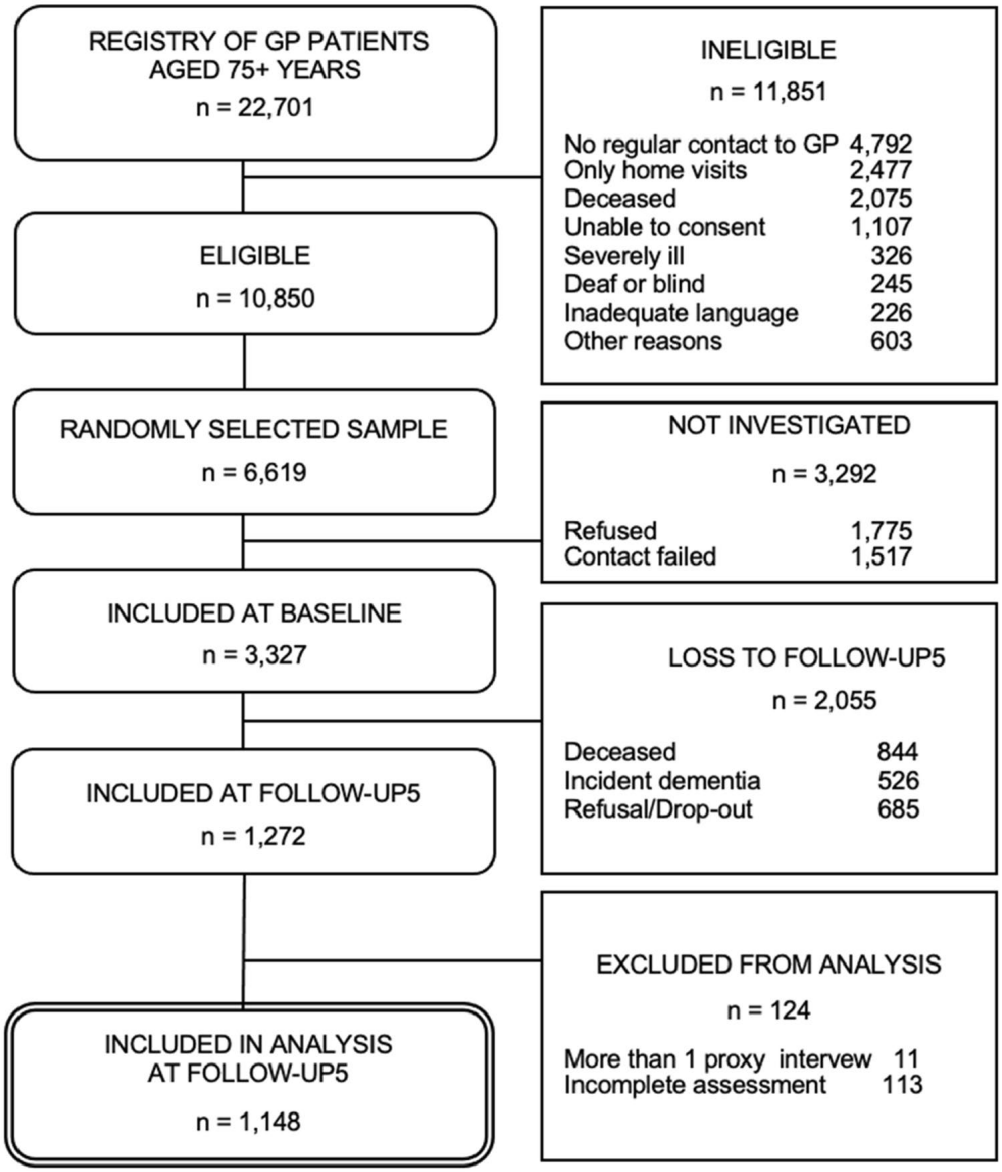
Flowchart of analytical sample at baseline

A flowchart of sample selection is shown in Fig. 1. A total of *N* = 3327 participants gave written informed consent and were included in the baseline sample (Fig. 1). Up to follow-up wave 5, *n* = 844 (25.4%) participants deceased, *n* = 526 (15.8%) had developed dementia and *n* = 685 (20.6%) refused or dropped-out due to other reasons. Of the remaining *n* = 1272 participants included in follow-up 5, *n* = 124 (9.7%) had to be excluded from the analyses due to incomplete assessments or because they had more than one proxy interview in the previous follow-ups. The resulting analytical sample consisted of *n* = 1148 participants.

### Assessments

#### Social isolation

Social isolation was assessed using the Lubben Social Network Scale 6 (LSNS-6), which examines the social network and the quality of social support of older individuals. The LSNS-6 is a statistically well evaluated six-item, self-report questionnaire [21]. The measurement displays frequency, size, and closeness of contacts in the respondent’s social network. Each LSNS-6 question is scored on a 0–5 Likert scale, the total score is an equally weighted sum of the six items, with a total score between 0 and 30. A cut-off score of 12 points was established for the LSNS-6, with respondents scoring lower being defined as socially isolated [21, 22]. The LSNS-6 is set up in two times three questions, in which each set of three questions form two subscales for social isolation from family (e.g., “How many relatives do you see or hear from at least once a month?”) and friends (e.g., “How many friends do you feel close to such that you could call on them for help?”). The two subscales of family and friends are statistically well justified with high levels of internal consistency and stable factor structures [21]. Cut-off scores for the two subscales family and friends were validated at score 6 to best discriminate between isolated and not isolated people; respondents with a score of less than 6 points are defined as socially isolated with regard to the respective context.

In an explorative factor analysis in our study, the two-dimensional structure of the LSNS-6 with the family and friends subscales was confirmed. The eigenvalues suggested strong principal components. Initial eigenvalues indicated that the first two factors explained 38.8% and 33.2% of the variance, respectively. Factor 1 represented the LSNS-6 family subscale. These items had primary factor loadings ranging from 0.87 to 0.88. Factor 2 represented the LSNS-6 friend’s subscale. These items had primary factor loadings ranging from 0.79 to 0.82. The item rest correlation for the two subscales family and friends ranged between 0.71 and 0.73 and 0.54 and 0.58, respectively. The resulting overall Cronbachs alpha for the family and friends subscales was α = 0.85 and α = 0.75, respectively.

#### Determinants

To cover potential risk factors of social isolation, a number of determinants were collected including sociodemographics, somatic and psychological aspects. Data on these determinants was provided in a standardized interview and by patient’s GPs.

Sociodemographic determinants included age, gender, marital status (married vs. not married/widowed/divorced), living situation (living alone vs. not alone), children (yes vs. no) and level of education. To constitute the determinant of education, the CASMIN classification with the educational level of low, medium or high was chosen [23]. Due to sparsely populated cells, medium and high education were collapsed into one category.

Furthermore, a number of health-related determinants were considered as risk factors of social isolation. First, impairments in vision, hearing and mobility were assessed as self-reported ratings and categorized as indicator variables (impaired vs. not impaired). To collect the data on somatic comorbidities, the general physician completed a questionnaire regarding the health of the participants. The following somatic comorbidities were requested: cardiac diseases, insult, hypertension, kidney insufficiency, diabetes mellitus, atherosclerosis, Parkinson's disease and illnesses of the thyroid gland. For simplicity, we used this information to generate a determinant indicating the number of somatic comorbidities in a patient (range 0–8). In addition, we used the Mini-Mental State Examination (MMSE) test to screen for cognitive functioning [24]. The MMSE results in a sum score ranging from 0 to 30, with higher scores indicating better cognitive performance. Moreover, we assessed depressive symptoms with the Geriatric depression scale (GDS), a self-report assessment consisting in its short form of 15 items (GDS-15). The GDS-15 is a reliable and valid screening instrument for detecting depressive symptom severity in elderly people, as well being sensitive to depression among elderly people suffering from mild to moderate dementia and physical illness [25].

Finally, difficulties in instrumental activities of daily living (IADL) were assessed via the 8-item Lawton and Brody IADL scale [26]. The analyses included only data on the five items common to both males and females, excluding the frequently female-associated items of food preparation, housekeeping and laundry. The resulting total score ranged from 0 to 5 points, with higher scores indicating less difficulties in activities of daily living.

### Statistical analysis

Sociodemographic and health-related determinants as well as figures on the prevalence and distribution of social isolation are reported as means with standard deviation or frequencies with percentages, as appropriate. Gender differences in social isolation were tested using Pearson chi-square tests for prevalence variables and *t* tests for LSNS-6 scores. Multivariate linear (OLS) regression models were conducted to test the association of sociodemographic and health-related determinants with social isolation related to family and friends, as measured by LSNS-6 subscales. The assumptions for the linear model were tested and confirmed psychometrically.

All statistical analyses were performed using STATA 16.0 SE (Stata Corp LP, College Station, TX) and used an alpha level of 0.05 (two-tailed) for statistical significance. The clustered design of the study was accounted for by adjusting the standard errors in the regression models using the practice ID as a cluster variable.

## Results

### Sample characteristics

Sample characteristics are shown in Table 1. The mean age of the analytical sample of 1,148 participants was 86.6 years (SD 3.0). Two in three participants were female (67.3%). The majority of participants were unmarried or widowed, was living alone, had children and low school education. With regard to physical impairments, the majority of participants reported walking difficulties (60.5%), almost half (48.8%) had a hearing impairment and a quarter (26.3%) had visual impairment. On average, participants had normal cognition (MMSE mean score 27.8, SD 1.9), hardly had any deficits in activities of daily living (IADL mean score 4.2, SD 1.1) and no signs of depression (GDS-15 mean score 2.6, SD 2.5). Moreover, participants reported on average 2–3 somatic comorbidities, with arterial hypertension (86.0%) and cardiac diseases (55.4%) being reported most frequently.

**Table 1:** Distribution of sociodemographic and health-related determinants

	Total
	*N* = 1148
Sociodemographic	
Gender, *n* (%)	
Male	376 (33.7)
Age, mean (SD)	86.6 (3.0)
Marital status, *n* (%)	
Unmarried/widowed	806 (70.2)
Married	342 (29.8)
Level of education, *n* (%)	
Low	643 (56.0)
Medium/high	505 (44.0)
Living situation, *n* (%)	
Living alone	638 (55.6)
Living with spouse/relatives	510 (44.4)
Having children, *n* (%)	931 (81.1)
Health-related	
Difficulties walking, *n* (%)	694 (60.5)
Sensory impairment, *n* (%)	
Vision	302 (26.3)
Hearing	560 (48.8)
MMSE, mean (SD)	27.8 (1.9)
IADL, mean (SD)	4.2 (1.0)
Depressive symptoms, mean (SD)	2.6 (2.5)
Somatic comorbidities, *n* (%)	2.4 (1.3)

*SD* standard deviation, *MMSE* mini-mental state examination, *IADL* instrumental activities of daily living

### Prevalence and distribution of social isolation

Overall, one in three participants was socially isolated (34.6%). Participants reported more frequent isolation from friends (44.3%) than from their family (21.8%). Although females had significantly lower mean scores than males on the LSNS-6 total and the friend’s subscale, there were no gender differences in these prevalence figures of social isolation (Table 2).

**Table 2:** Prevalence and distribution of social isolation (total, family, friends) according to LSNS-6

	Total		Gender			
	*N* = 1.148		Female (*n* = 772)		Male (*n* = 376)	
	*n* (%)	Mean (SD)	*n* (%)	Mean (SD)	*n* (%)	Mean (SD)
Prevalence						
Total, cut-off < 12	397 (34.6)		277 (35.9)		120 (31.9)	
Friends, cut-off < 6	508 (44.3)		345 (44.7)		163 (43.4)	
Family, cut-off < 6	250 (21.8)		185 (24.0)		65 (17.3)	
Distribution						
Total score		14.0 (5.58)		13.7 (5.4)*		14.6 (5.9)
Friends subscore		6.0 (3.8)		7.8 (3.6)*		8.3 (3.3)
Family subscore		8.0 (3.5)		5.9 (3.7)		6.2 (4.0)

*T* test or Chi2 test for comparison between females and males

*SD* standard deviation, *CI* confidence interval

*p < 0.05, **p < 0.01 ***p < 0.001

### Determinants of social isolation related to family

Analysis of determinants of social isolation related to family showed that males were significantly more socially isolated than females (β = − 0.68, 95% CI − 1.08, − 0.28; Table 3). In comparison, participants who reported being married were less socially isolated than those who reported being widowed or divorced (β = 0.96, 95% CI 0.22, 1.70). Moreover, lower education (β = 0.42, 95% CI 0.08, 0.76) and having children (β = 3.21, 95% CI 2.64, 3.78) were depicted as protective factors for social isolation related to the family. With regard to health-related determinants, impairment in walking had a significant impact on social isolation (β = − 0.42, 95% CI − 0.84, − 0.01), whereas better cognitive functioning was a protective factor against social isolation related to the family (β = 0.11, 95% CI 0.01, 0.22). Finally, depressive symptom severity was significantly associated with more social isolation in the family context (β = − 0.28, 95% CI − 0.36, − 0.20).

**Table 3:** Linear Regression of social isolation in the context of family and friends

	Family		Friends	
	*N* = 1148		*N* = 1148	
	*B*	95% CI	*B*	95% Cl
Sociodemographic				
Gender				
Male	− 0.68***	[− 1.08, − 0.28]	− 0.09	[− 0.57, 0.39]
Age	− 0.01	[− 0.08, 0.06]	− 0.12**	[− 0.19, − 0.05]
Marital status				
Married	0.96*	[0.22, 1.70]	0.54	[− 0.19, 1.26]
Level of education				
Low	0.42*	[0.08, 0.76]	− 0.51*	[− 0.93, − 0.09]
Living situation				
Living alone	− 0.56	[− 1.15, 0.03]	0.53	[− 0.03, 1.09]
Having children	3.21***	[2.64, 3.78]	− 0.08	[− 0.69, 0.53]
Health-related				
Difficulties walking	− 0.42*	[− 0.84, − 0.01]	0.27	[− 0.24, 0.78]
Sensory impairment				
Vision	− 0.32	[− 0.70, 0.06]	− 0.04	[− 0.51, 0.44]
Hearing	0.01	[− 0.30, 0.31]	0.22	[− 0.22, 0.67]
MMSE	0.11*	[0.00, 0.22]	0.22***	[0.11, 0.34]
IADL	− 0.20	[− 0.40, 0.00]	0.36**	[0.09, 0.64]
Depressive symptoms	− 0.28***	[− 0.36, − 0.20]	− 0.30***	[− 0.39, − 0.20]
Somatic comorbidities	0.01	[− 0.11, 0.13]	− 0.07	[− 0.23, 0.08]
*R*^2^	0.24		0.12	

*B* unstandardized regression coefficient, *CI* confidence interval, *MMSE* Mini-Mental State Examination, *IADL* instrumental activities of daily living

### Determinants of social isolation related to friends

Analyses of determinants of social isolation related to friends yielded somewhat different results. First, it was found that social isolation from friends was associated with higher age (β = − 0.12, 95% CI − 0.19, − 0.05) and lower education (β = − 0.51, 95% CI − 0.93, − 0.09). With regard to health-related determinants, results further indicated that less difficulties in handling daily activities were associated with less social isolation from friends (β = 0.36, 95% CI 0.09, 0.64). In addition, similar to the family context, cognitive functioning (β = 0.22, 95% CI 0.11, 0.34) proved protective against social isolation in relation to friends, while depressive symptom severity appeared to be associated with more social isolation (β = − 0.30, 95% CI − 0.39, − 0.20; Table 3).

## Discussion

### Main findings

This study aimed to estimate the prevalence of social isolation in the oldest-old as well as to examine the impact of multiple determinants on social isolation in relation to family and friends. We found that about one in three adults over 80, regardless of gender, were socially isolated, with isolation from friends being reported twice as often as isolation related to the family.

We also found that differences in the social context of isolation were predominantly determined by demographics, while health-related determinants were largely associated with social isolation in general, regardless of its context.

Our study adds significantly to the evidence for the high prevalence of social isolation in old age by expanding the age group studied to include the oldest-old. In previous population-based studies, the prevalence of social isolation in Germany was assessed only up to the age of 79 years, ranging between 12–13% before the COVID-19 pandemic [27, 19]. In our sample of 80+ year-olds, the prevalence of social isolation was more than twice as high at 34.6%. When distinguishing by context, we further found considerable differences in the prevalence of social isolation in relation to friends (44.3%) and family (21.8%). This underlines that social isolation in old age is a serious concern and there is a need to establish context-specific interventions to reduce social isolation especially in relation to friends in order to maintain health long-term.

We found several differences in the severity of social isolation from family and friends related to sociodemographic characteristics. First, in the multivariate analysis, male gender was associated with social isolation in the family, but not in friends. This is consistent with a study indicating that males are significantly more socially isolated in the family than in friends [28]. In general, the social networks of males and females may differ in that females have a larger social network and tend to be more satisfied with their network than males. Moreover, with increasing age the size of the social network diminishes substantially [13], and this was more prone in males than in females [29]. The gender difference may affect the consequences of social isolation as well. According to a population-based 18-year follow-up study from the US, social isolation has a greater impact on chronic illnesses and mortality in males than in females, possibly due to a heightened inflammatory response to the disruption of social ties [30]. Altogether, older males with their smaller social network seem more vulnerable to social isolation and its consequences, especially when related to the family.

Although age is regarded as one of the main risk factors for social isolation [31], our results indicated that this is only true in relation to friends. This could indicate changes and transitions in the social network of older adults from friends to family as they grow older. This is corroborated by the findings of Wrzus et al. [32] showing a decrease in the network size of friends, whereas the family network size remains stable. However, conflicting findings [28] show that age as a risk factor for social isolation is more complex than previously thought. Further research considering the social context of isolation among older adults is needed to unravel the role of aging in older adults’ family and friends networks.

Marital status is often considered in research on social isolation, as it provides a first insight into the everyday social interactions of respondents. The protective effect of marriage on family-related social isolation that we found is not surprising and has been confirmed more generally in several studies [33]. Likewise, having children also protected against isolation in the family context, certainly due to the emotional, physical, and financial support that (adult) children can provide [34]. What is interesting, though, is that marriage did not play a substantial role in social isolation from friends. Moreover, living alone was not associated with social isolation, neither related to family nor to friends. Despite possible health restrictions, married or cohabitating people seem to focus no less on friends (in favour to their own partnerships) than people who are widowed or live alone do.

Another interesting, yet contrastive finding for social isolation related to family and friends was found with regard to educational attainment. A low level of education was protective against social isolation related to the family, whereas it was a risk factor for social isolation related to friends. This complements previous studies suggesting that low education is associated with higher levels of social isolation in general [35]. It may be that the family environment is generally more inclusive and tolerant, while education promotes protection against isolation from friends, particularly in older age.

We also found evidence for several health-related determinants of social isolation, with the results often being similar in the family and friends' contexts. First, cognitive functioning was associated with lower social isolation related to both family and friends. This complies with other studies showing that dementia impairs communication and social interactions, eventually leading to social isolation [27]. In addition, reverse causation is also conceivable: socially isolated individuals have less social interaction and therefore receive less cognitive stimulation, ultimately resulting in cognitive decline [36]. Taken together, the results underline the importance of the association between cognition and social isolation, which in our study appeared to be even more pronounced in the context of friends than in the family.

Next, depressive symptoms were a strong determinant of social isolation both from family and friends, lining up with more general studies on depression and social isolation [4]. Depression is not only a frequent result of declining health and functional impairment in older adults [37] but also an important correlate of loneliness and social isolation [4]. The relationship between depression and social isolation is complex; some claim it is sequential [4], some say it is likely to be reciprocal [38].

Our study further revealed an association of difficulties in instrumental activities of daily living (IADL) with social isolation from friends. Restrictions in IADL appear to pose a serious obstacle to social contacting and participation outside the family. Interestingly, another study [28] found the opposite, reporting that impairments in IADL were associated with less isolation from friends. The authors assumed that an increased need for care entails more social interactions with caregivers, which ultimately leads to more friendships. However, participants in our study were on average 20 years older and more often widowed/living alone than in the mentioned study. It is very likely that the particular role of difficulties in daily care routines substantially affects the social network with increasing age.

Contrary to intuition, walking difficulties were another independent predictor of social isolation in the family but not in the friend’s context. The inability to participate in family social gatherings due to physical disabilities may be associated with a kind of (unwanted) self-isolation. In contrast, activities in the friends’ context could be planned better and more specifically despite existing physical disabilities.

Unlike other studies looking at sensory impairments and somatic comorbidities [39], we did not find significant associations with social isolation in relation to family or friends. One explanation could be that we did not consider the severity of disabilities in our study; a more severe and clinically relevant condition is more likely to be associated with social isolation, regardless of context. In addition, the selection of examined diseases may have been too small, since various somatic comorbidities undoubtedly differ in their degree of impairment and limitation. A broader range, including neurological and cancer diseases and information on the extent of medical control should be included in further research.

### Limitations

Our study was not without limitations. First, data were collected prior to the Covid-19 pandemic. Due to social distancing, quarantine measures and decreasing social interaction, increasing levels of loneliness and social isolation were found among all age groups during the pandemic [40]. One study found a perceived isolation of even 59% in the age group between 18 and 70 years [41], almost five times as high as the mentioned range of 12–13% found prior to the COVID-19 pandemic [19, 27]. Changes in determinants, prevalence and patterns of social isolation post-pandemically remain unclear in this study. Second, an inherent selection bias in our analyses cannot be ruled out with certainty, as some participants could not be contacted, were excluded from the data due to lack of information, or declined participation in the beginning or during the follow-up studies. The analyses may particularly omit more severely ill cases since these participants either did not meet the inclusion criteria or have already dropped out in the first four waves of the cohort study. Third, data were drawn only from self-administered questionnaires, which is prone to reporting bias. Since social isolation is possibly attached to stigma, individuals may underreport social isolation and the prevalence may be underestimated. Our aim was to consider as many determinants of social isolation as possible, but the selection of determinants was limited to those chosen during the planning and conduction process of the study. There may be more determinants, such as income and demographic factors (e.g. metropolitan vs. non-metropolitan) [42], which have not yet been assessed. Finally, due to the cross-sectional design, our study cannot conclude causality. A longitudinal approach is required to depict developments in the social network of older people, e.g. after critical life events such as the loss of a partner or hospitalization.

## Conclusion

Given the multitude of health implications, preventing social isolation in old age should be of paramount health and societal concern [43–45]. With a 35% prevalence of social isolation among people aged 80+, the urgency to address this issue is particularly evident. Due to demographic change and the long-term effects of the COVID-19 pandemic, the importance of the social integration of older people into society might increase even more in the future. Our study further provides evidence of contextual and independent factors influencing social isolation. Older males with low education, cognitive impairment and depressive symptoms are particularly at risk and should receive special attention in terms of prevention. Initiatives such as intergenerational programs, making communities more age-friendly, or facilitating access to services and public spaces for the elderly population could help prevent social isolation, improve quality of life and health in old age, and avert psychological and physical harm [44, 46].

## Data Availability

The data used and analyzed in this study can be made available to researchers on reasonable request to the correspondent author.
